# Research on High-Efficiency and No-Additive Physical Aging Equipment and Process of Baijiu Production Based on High-Speed Jet Catalysis

**DOI:** 10.3390/foods14234019

**Published:** 2025-11-24

**Authors:** Zhongbin Liu, Fengkui Xiong, Guangzhong Hu, Hongwei Xiao, Jia Zheng

**Affiliations:** 1 College of Mechanical Engineering, Sichuan University of Science & Engineering, Yibin 644001, China; 2 College of Engineering, China Agricultural University, Beijing 100080, China; 3 Wuliangye Group Co., Ltd., Yibin 644001, China

**Keywords:** physical aging of liquor without addition, high efficiency, high speed jet cavitation, CFD modeling, research and development of technical equipment

## Abstract

Newly brewed Baijiu often contains harmful substances such as mercaptan and methanol, which are spicy and harmful to health. At present, this is mainly solved by long-term cellaring, but this is faced with some problems such as a long cycle, high cost, high fire hazards and so on. Therefore, based on the principle of liquid jet cavitation explosion catalyzing the heterogeneous association of Baijiu molecules, this paper first developed the physical aging process and equipment without radiation and additives. Then, based on the traditional computational fluid dynamics (CFD) model of high-speed jet simulation, an N-CFD model which can accurately simulate the cavitation explosion catalytic process of high-speed jet of Baijiu was established by optimizing the three sub models of conservation, turbulence and VOF. Finally, the N-CFD model was used to optimize the distance between the nozzle and the reaction chamber wall of the new aging equipment. Through the 15 min aging experiment on 100 L Baijiu, the methanol concentration of Baijiu decreased by 68.14 ± 2.25%, and the concentration of ethyl acetate, ethyl lactate and ethyl palmitate increased from 6.105 ± 0.014, 3.498 ± 0.015 mg/L and 0.621 ± 0.010 mg/L to 6.332 ± 0.016, 4.868 ± 0.012 mg/L and 0.681 ± 0.008 mg/L. The results show that the aging technology equipment can adjust the self-coupling characteristics and dynamic characteristics of various molecules in Baijiu through high-speed jet, and catalyze the alternating phase transition and association of various molecules. Finally, the goal of high-efficiency and healthy aging Baijiu without additives was achieved, which helps the rapid and healthy development of the Baijiu brewing industry.

## 1. Introduction

As a cultural element, Baijiu has permeated the entire Chinese civilization. It is not only a gem of Chinese culture but also one of the pillars of the national economy, presenting a “hundred-flower-blooming” trend across the country [1,2]. However, newly brewed Baijiu contains more hydrogen sulfide and methanol, which leads to its spicy taste and strong irritation [3,4]. At present, the traditional aging process of “cellaring + ceramic jar storage”, which takes more than 3 years, is widely used in Baijiu production [5,6,7]. Although the process can slowly associate ethanol, acetic acid and other substances to make the Baijiu aromatic and pure through long-term storage, there are still problems to be solved such as high capital construction cost, high capital backlog cost and high fire risk. Therefore, the development of efficient, non-additive and healthy Baijiu aging technology and equipment to replace the traditional aging process has become one of the main topics of interest in the Baijiu brewing industry.

The discussion on the Baijiu aging mechanism led by the State Council originated in the 1960s. So far, artificial aging hypotheses such as “association theory”, “esterification theory”, “oxidation theory”, “dissolution theory” and “volatilization theory” have been put forward [8,9,10]. According to disciplines, it can be divided into three categories: physical aging, chemical aging and biological aging [11,12]. Chemical aging is to accelerate the chemical reaction between different components in Baijiu by adding ozone, metal ions and other chemical catalysts, so as to accelerate the generation of esters and other flavor substances [13]. Biological aging is the biochemical reaction between specific microorganisms and substances in Baijiu to produce aromatic substances and absorb harmful substances such as fusels, so as to improve the taste and flavor of Baijiu [14,15,16]. However, the chemical aging method requiring the addition of a catalyst and the biological aging method requiring the addition of microorganisms cannot be recognized by consumers, nor can they meet the requirements of food safety regulations, so they are not put into production on a large scale. Traditional physical aging is a technology that uses radiation, magnetism and other functions to catalyze the alternating phase transition and association of various molecules in Baijiu to promote the rapid aging of Baijiu [17,18,19]. Although there are no exogenous additives, there are also inherent defects such as harm to the human body and high equipment cost, hindering production on a large scale. To sum up, currently, there is still a lack of high-efficiency, non-additive and harmless pure physical aging technology and equipment that can be applied to the large-scale production of Baijiu, which seriously restricts the development of the Baijiu industry. Therefore, the development of a set of pure physical aging technology equipment for Baijiu, which takes into account high efficiency, health, no additives and low cost, is essential for the low-cost, high-efficiency and high-quality development of the Baijiu industry.

Therefore, based on the extreme changes in pressure, flow field and temperature in the process of high-pressure high-speed jet through small holes, this paper will describe the utilization of cavitation and violent explosions, which catalyze the high-speed reaction of water, ethanol, acetic acid, lactic acid and other components in Baijiu. First of all, we developed a pure physical aging process and equipment for Baijiu with no additives, no harm, at a low cost and high efficiency. Then, based on the traditional CFD model of high-speed jet simulation, an N-CFD model which can accurately simulate the cavitation explosion catalytic process of the high-speed jet of Baijiu was established by optimizing the three sub models of conservation, turbulence and VOF. Then, the N-CFD model was used to optimize the distance between the nozzle and the reaction chamber wall of the new aging equipment. Finally, through the application and comparative detection in “Wuliangye Group Co., Ltd.”, “Jinliangzao Group Co., Ltd.” and other enterprises, the efficiency, health and advancement of the new physical aging process equipment were verified, so as to solve the bottleneck problem restricting the rapid development of the Baijiu industry.

## 2. Theoretical Design of the Physical Aging Process and Equipment

### 2.1. Design of Aging Process

#### 2.1.1. Jet Cavitation Mechanism

The cavitation degree of Baijiu can be represented by the dimensionless cavitation number as follows:

(1) $$\sigma =\frac{2(P_{0}-P_{v})}{\rho \nu_{0}^{2}}$$

In Equation (1): $P_{0}$—Ambient pressure; $P_{v}$—Saturated vapor pressure of the wine body; $v_{0}$—Nozzle exit velocity; and ρ—Density of the wine body.

The transition of the cavitation state has a significant critical point. When the wine body leaves the impeller and enters the nozzle, the flow velocity suddenly increases twice (*V∞*), which can instantaneously reduce the cavitation number below the incipient cavitation number ($\sigma_{i}$), resulting in the generation of a large number of cavitation bubbles. Immediately afterwards, when the wine body forms a high-speed jet after leaving the nozzle, the sudden decrease in flow velocity and the sudden increase in pressure when it collides with the cylinder wall can instantaneously raise the cavitation number ($\sigma_{d}$) above the extinction cavitation number, causing the cavitation bubbles to violently burst. Meanwhile, the cavitation hysteresis effect caused by the usually unequal characteristics of $\sigma_{i}$ and $\sigma_{d}$ can also enhance the bursting of cavitation bubbles. In summary, the generation, development, and bursting processes of cavitation bubbles in the flow field are extremely complex and are greatly affected by pressure and flow velocity in fluid dynamics.

Current research generally suggests that the jet cavitation number, the initial physical and chemical properties of the solution, and the characteristics of dissolved gases are the key factors determining the intensity of jet cavitation. During the physical aging process of Baijiu, the characteristics of dissolved gases in the Baijiu often depend on the air conditions during brewing and the physical aging treatment, rather than the aging process. Therefore, in the physical aging process of Baijiu based on the jet cavitation effect, the jet cavitation number and the physical and chemical properties of the Baijiu are the key factors determining the aging effect of Baijiu.

(1) Jet cavitation number

For the Baijiu jet to produce a chemical effect, it is necessary that the Baijiu is fully cavitated during the jet process, forming a sufficient number of cavitation bubbles with an appropriate degree, which then burst violently. The jet cavitation number, a dimensionless parameter used to measure the degree of jet cavitation, is closely related to the jet cavitation intensity. Increasing the nozzle exit velocity or reducing the ambient pressure can decrease the jet cavitation number, thereby enhancing the degree of jet cavitation. However, the ambient pressure needs to be maintained at a reasonable level. If the ambient pressure is too low, the temperature and pressure generated instantaneously during the collapse of cavitation bubbles will also decrease, ultimately reducing the effectiveness of the chemical effect of the cavitating jet. Therefore, how to design and optimize the nozzle structure to maximally meet the cavitation requirements for the physical aging of Baijiu is undoubtedly the key and difficult point of this study.

(2) Physicochemical properties of the wine body

Current research suggests that the initial physicochemical properties of the Baijiu body, especially the alcohol content, are crucial for optimizing the process conditions of cavitation aging and exploring the reaction kinetics laws. Moreover, it is the core factor determining the aging effect of Baijiu [20,21]. Experimental studies by Jiang et al. [22] have shown that as the initial alcohol content of Baijiu increases, the catalytic effect (aging speed) of ultrasonic cavitation significantly decreases. The reason may be that the increase in alcohol content leads to an increase in solute vapor inside the cavitation bubbles, resulting in a decrease in temperature when the cavitation bubbles collapse, thus slowing down the reaction rate. However, Huang et al. [23] reached the opposite conclusion through CFD simulation analysis. Therefore, current research on the influence of alcohol content on the cavitation catalytic effect and its mechanism is still controversial and urgently needs to be resolved.

#### 2.1.2. Mechanism of Heterogeneous Association Catalytic Reaction

**Figure 1:**
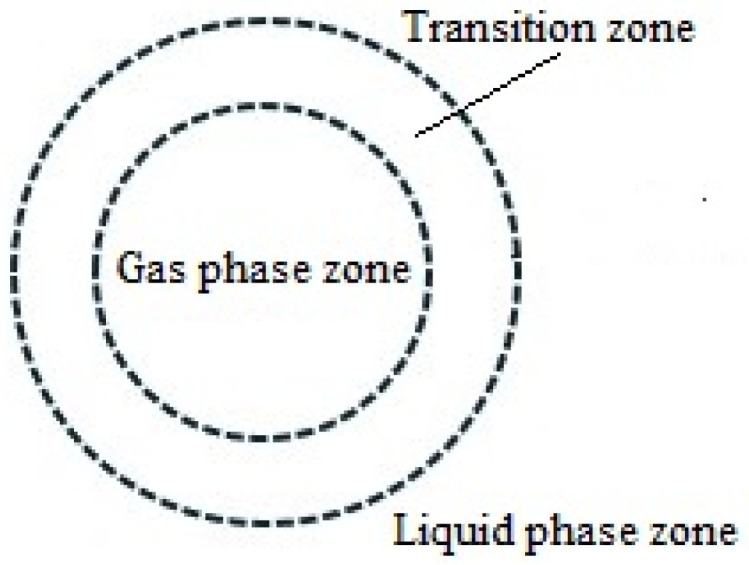
Schematic diagram of vacuole partitioning.

The rapid and violent explosion of cavitation bubbles formed by jet cavitation in the wine body is the fundamental cause for catalyzing the association reaction among various components in the wine body. The process of the rapid and violent explosion of cavitation bubbles can be subdivided into three parts according to the effect of catalyzing the association reaction: the gas-phase region, the transition region, and the liquid-phase region, as shown in Figure 1.

(1) Bubble zone

Under the extreme conditions of intense cavitation bubble collapse, a special reaction zone composed of water vapor, readily soluble gases, and high-pressure vapors of volatile solutes is formed inside the cavitation bubble. At the moment of intense bubble collapse, the instantaneous temperature and pressure in this zone can reach 5200 K and 50 MPa, respectively. Theoretically, being in the extreme environment of high temperature and high pressure during the collapse, it is bound to catalyze the direct thermal decomposition reaction of water vapor and non-polar volatile solutes and oxidation reactions initiated by various free radicals.

(2) Transition zone

During the violent explosion of the cavitation bubble, there is a superheated liquid layer (i.e., the bubble wall) with a thickness of approximately 200 nm surrounding the bubble region, which we define as the transition zone. During the violent explosion, the temperature in this region can instantaneously soar to 1900 K, causing the water in this region to assume a supercritical state and the solute concentration to increase rapidly. Theoretically, it can catalyze the oxidation reaction between polar, non-volatile solutes in this region.

(3) Liquid Phase Region

In the entire wine body, the liquid phase is its main component and is an ordinary region where the temperature and pressure show no obvious changes and are closest to the environmental conditions. Therefore, theoretically, this region mainly serves to receive the strongly oxidizing substances remaining after the reactions in the bubble region and the transition region and provides abundant solutes, with relatively slow reactions.

#### 2.1.3. Process Scheme Design

**Figure 2:**
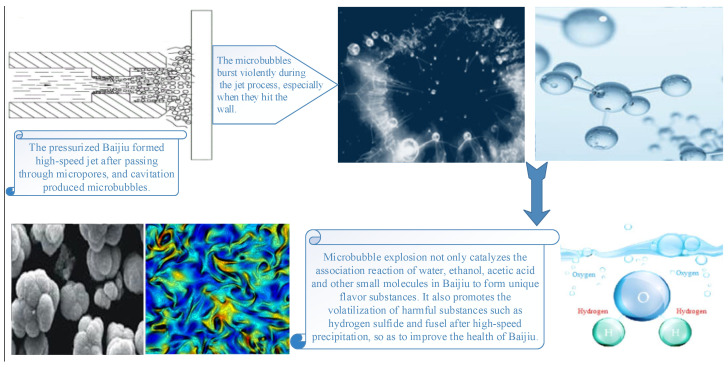
Schematic diagram of the physical aging process of Chinese Baijiu.

Based on the cavitation principle of high-speed liquid jets, the shape and size of the nozzle and its distance from the cavity wall are precisely controlled to generate a large number of cavitation bubbles during the jetting process of Baijiu. These bubbles undergo intense implosion during the jetting, especially at the moment of impacting the cavity wall. Through the instantaneous and intense implosion of the cavitation bubbles, the association reactions of small molecules such as water, ethanol, acetic acid, and lactic acid in Baijiu are catalyzed to enhance the formation of unique flavor compounds. Meanwhile, the rapid precipitation and volatilization of harmful substances such as hydrogen sulfide and fusel alcohols are promoted to improve the health-friendly level of Baijiu. Ultimately, harmless, efficient, and high-quality aging of newly brewed Baijiu is achieved. The process flow is shown in Figure 2.

As shown in Figure 2 above, the technological process of high-efficiency and high-quality physical aging of Baijiu is as follows. First, under the action of the vacuum environment generated by the high-speed rotation of the impeller and the strong centrifugal force, Baijiu is sucked from the wine storage barrel into the cavity and is thrown at high speed to the nozzle inlet at the edge of the cavity. Then, under the combined action of the high pressure formed by the high-speed Baijiu flow and the strong suction of the vacuum in the cavity at the nozzle outlet, Baijiu is accelerated again to pass through the nozzle with a specific narrow shape (d ≤ 10 mm), and an extremely high-speed jet is formed at the nozzle. Meanwhile, under the combined action of the overall high pressure of the extremely high-speed jet and the local low pressure on the solid–liquid contact surface, the air and wine vapor dissolved in the Baijiu form cavitation bubbles. Second, during the jet process, especially when impacting the cavity wall, the cavitation bubbles burst violently in an instant. Along with the instantaneous high-temperature and high-pressure changes caused by the violent bursting of the cavitation bubbles, the chemical equilibrium in the Baijiu is broken, thus catalyzing the association reaction between multi-phase molecules in the Baijiu. Finally, the goal of high-efficiency, high-quality, additive-free and harmless aging of Baijiu is achieved.

### 2.2. Structural Design of Aging Equipment

#### 2.2.1. Overall Structure Design

**Figure 3:**
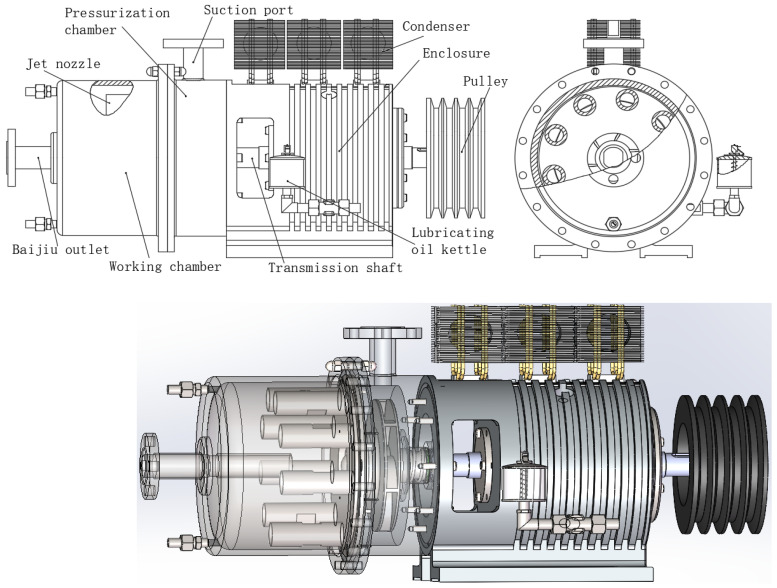
Schematic diagram of the structure of the new physical aging equipment.

Based on the above analysis of the mechanism of the multiphase association reaction in Baijiu catalyzed by high-speed jet, it is not difficult to conclude that the physical aging process of Baijiu mainly consists of six procedures: Baijiu suction and pressurization by the impeller, high-speed jet, Baijiu cavitation, bubble collapse, Baijiu extrusion, and condensation and cooling. The corresponding pure physical aging equipment for Baijiu should include seven basic systems: power system, impeller, pressurization chamber (including Baijiu suction port), nozzle, reaction chamber, Baijiu outlet, and condenser. Adding typical connecting components based on these seven systems, the overall structure of the physical aging equipment for Baijiu is developed as shown in Figure 3.

As shown in Figure 3, the aging process of Baijiu in this new equipment is as follows: ① Baijiu is connected from the storage tank to the pressurization chamber through the liquid inlet pipe and the wine suction port. The high-speed rotating impeller forms a negative pressure area in the pressurization chamber, sucking Baijiu and air simultaneously and mixing them thoroughly. ② The strong centrifugal force generated by the high-speed impeller throws the mixed flow of Baijiu and air at high speed to the outer ring wall of the pressurization chamber, and then it enters the nozzle at high pressure and high speed. ③ Under the action of speed increase and pressurization in the narrow flow channel of the nozzle, the mixed flow of Baijiu and air breaks through the cavitation number limit and generates a large number of cavitation bubbles. ④ After leaving the nozzle, Baijiu immediately forms a jet. ⑤ During the jet process, especially when impacting the wall of the reaction chamber at a specific distance, the cavitation bubbles burst violently in an instant. Along with the instantaneous high-temperature and high-pressure changes during the violent bursting of cavitation bubbles, the chemical equilibrium in the wine is broken, thereby catalyzing the association reaction between multiphase molecules in the wine. ⑥ The wine flows out through the wine outlet, is cooled by the condenser, and then returns to the Baijiu storage tank along the pipeline. Through several cycles of Baijiu in the wine storage tank, 50–500 kg of Baijiu can be processed in a single cycle that takes 15 min, achieving the goal of high-efficiency aging of Baijiu.

In summary, the structures of the jet nozzle and the reaction chamber jointly determine the intensity of the jet cavitation effect and the degree of cavitation bubble explosion, and they are the key components that determine the effect of physical aging of Baijiu. Therefore, optimizing the structures of the jet nozzle and the reaction chamber through “theory + CFD simulation” design is crucial to ensure that their jet cavitation effect can best catalyze the multiphase association and oxidation reactions in Baijiu, which is the key to achieving the goal of efficient and additive-free Baijiu aging.

#### 2.2.2. Theoretical Design of the Jet Nozzle and Reaction Chamber Structure

**Figure 4:**
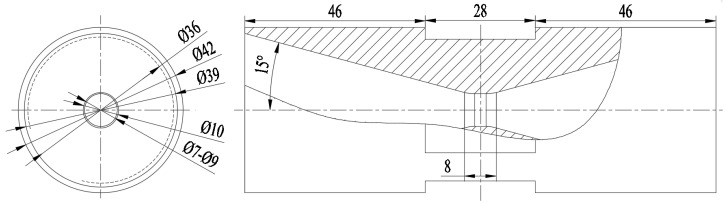
Structure of the jet nozzle.

According to the requirements of jet cavitation theory, the structure of the designed jet nozzle is shown in Figure 4. The jet nozzle is installed on the common wall of the pressurization chamber and the reaction chamber through a neck with a diameter of 42 mm.

The core reaction process of Chinese Baijiu aging mainly consists of two core procedures: accelerating and pressurizing, and decelerating and depressurizing. (1) Accelerating and pressurizing procedure: Under the combined action of the strong centrifugal force generated by the high-speed rotation of the impeller and the inner wall of the pressurizing chamber, Baijiu enters the inlet of a nozzle with a diameter of 39 mm at a high speed. Then, under the guidance of a 15° conical flow, it is further pressurized and reaches the spiral injection holes with an inner diameter d = 7–9 mm and an outer diameter D = 10 mm. Under the action of the continuously decreasing diameter of the drainage conical hole and the spiral of the injection holes, the flow velocity and pressure of Baijiu rise rapidly, breaking the limit of the cavitation jet index. A large number of cavitation bubbles are formed rapidly and tend to be stable under the action of the spiral turbulent flow. (2) Decelerating and depressurizing procedure: When Baijiu leaves the nozzle, the pressure and flow velocity decrease sharply. In particular, when it collides with the wall of the reaction chamber at a specific distance L, the super-strong impact causes the cavitation index to rise rapidly, leading to the instantaneous and violent explosion of the cavitation bubbles. The instantaneous changes in high temperature and high pressure caused by the instantaneous and violent explosion of the cavitation bubbles will break the chemical equilibrium in the Baijiu, thus catalyzing the association reaction between multi-phase molecules in the Baijiu.

In conclusion, since impellers are generally standard parts and only need to be selected according to the working conditions, the inner diameter d of the nozzle and the distance L from the reaction chamber wall to the nozzle determine the effect of the cavitation effect on catalyzing the aging of Baijiu. Therefore, this study will use CFD simulation analysis to optimize the inner diameter d of the nozzle and the distance L from the reaction chamber wall to the nozzle to complete the design of the aging equipment.

## 3. CFD Modeling and Optimization Design of Key Components

### 3.1. Physical Modeling and Boundary Condition Setting

**Figure 5:**
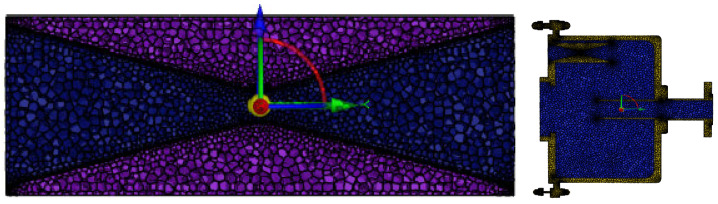
Schematic diagram of the nozzle and reaction chamber grids.

The physical models of the nozzle and the reaction chamber were established using SolidWorks 2024 and imported into the ICEM CFD module of Fluent for mesh generation as shown in Figure 5. The initial boundary conditions were set according to the actual working conditions as shown in Table 1.

### 3.2. Optimization of CFD Simulation Model

The CFD model mainly consists of two parts: the basic conservation equations and the fluid dynamics characteristic equations. Since the heat exchange between the Baijiu flowing in the nozzle and the air is negligible compared to the intense cavitation bubble explosion process, only the energy conservation equation during the intense cavitation bubble explosion needs to be considered in the modeling process [24]. Currently, the simulation analysis of multiphase flow jet processes, such as those of petroleum and Baijiu, is generally based on the traditional volume of fluid (VOF) model and the k-ε turbulence model. The accuracy of the VOF and turbulence models often determines the accuracy of cavitation simulation as well as the efficiency and precision of calculations. However, the traditional VOF model assumes that the velocities and pressures of the gas–liquid two phases are the same within the same grid and only solves a single momentum Eq, which weakens the resistance caused by the velocity difference between the gas and liquid phases at the phase interface and fails to meet the requirement of obtaining an accurate gas–liquid two-phase interface for the research on cavitation and cavitation bubble explosion. Meanwhile, the k-ε turbulence model is also unable to accurately simulate the large eddies formed by the jet impact disturbance in the reaction chamber. Therefore, this study will optimize the conservation model, turbulence model, and traditional VOF model based on the traditional simulation analysis method to accurately simulate and analyze the cavitation and cavitation bubble explosion processes.

#### 3.2.1. Optimization of the Conservation Sub-Model

Assuming that the physical property parameters such as the density and viscosity of Baijiu and its vapor are constant and the inlet pressures are the same, the three phases of the Baijiu body, Baijiu vapor, and air should follow the basic conservation equations composed of the continuity equation. and the momentum conservation equation. In this study, the mass source term generated by cavitation is added to the continuity equations of the Baijiu body and its vapor, and the effects of the drag force *F_d_*_,*i*_ and the surface tension *F_s_*_,*i*_ at the gas–liquid interface of the cavitation bubbles on the cavitation effect are fully considered. Then, the continuity equation and the momentum conservation equation of the conservation model can be optimized as follows, respectively. In all the formulas, the subscripts *i* and *j* represent the liquid phase and the gas phase in the Baijiu body, respectively.

(2) ∂(ρiαi)∂t+∇(ρiαiui)=mij

(3) ∂(ρiαiui)∂t+∇(ρiαiuiui)=∇[αiui(∇ui+∇uiT)−1.5kI]−αi∇p+mijuij+fvm,i+Fs,i+mijuij+fd,i

In Equations (2) and (3): *ρ*—density, kg/m^3^; *α_i_*—volume fraction; *u_i_*—velocity, m/s; *t*—time, s; m_ij_—mass source term; *F_vm_*_,*i*_—virtual mass force; *F_d_*_,*i*_—drag force, N; *F_s_*_,*i*_—surface tension, N; *p*—inlet pressure; $k_{p}$—turbulence energy coefficient; *I*—unit tensor. When *m_ij_* is positive or negative, *u_ij_* corresponds to *u_j_* or *u_i_*, respectively.

The drag force caused by the velocity difference between phases is the main inducement of jet cavitation. The expression of the drag force *F_d_*_,*i*_ is

(4) Fd,i=∑j=1j≠1nαiαj[3Cd,iρjui−uj(ui−uj)]4di

In Equation (4): *u_i_* − *u_j_*—the velocity difference between phases, m/s; *d_i_*—bubble diameter, mm; *C_d_*_,*i*_—drag coefficient. According to the calculation model of Schiller and Naumann, the following equation could be obtained:

(5) Cd,i=24(1+0.15Re0687)/Re0.44(Re≤1000)(Re>1000)

(6) Re=diui−ujvj

In Equations (5) and (6): *Re*—Reynolds number; *v_j_*—kinematic viscosity.

*F_s_*_,*i*_ is a crucial factor controlling the characteristics of the two-phase interface, which significantly impacts cavitation and the subsequent violent explosion. Its equation is as follows:

(7) $$F_{s,i}=\sum_{\begin{array}{l} j=1\\ j\neq 1 \end{array}}^{n}\sigma_{ij}\kappa (\alpha_{j}\nabla \alpha_{i}-\alpha_{i}\nabla \alpha_{j})$$

In Equation (7), $\sigma_{ij}$—the surface tension coefficient between the two phases; κ—the local interface curvature, which can be calculated by Equation (8).

(8) κ=∇(αj∇αi−αi∇αj)αj∇αi−αi∇αj

By substituting the above equations into the original conservation model in the Fluent module, the basic conservation equations that can accurately describe the aging process of Chinese Baijiu can be obtained.

#### 3.2.2. Optimization of Turbulence Sub-Model

Compared with the currently commonly used k-ε turbulence model, the Large Eddy Simulation (LES) model can accurately capture complex and inherently unsteady flow fields, thereby better simulating the large eddy phenomenon formed by the turbulent flow in the reaction chamber. However, LES has relatively high requirements for grid quality, and the grid resolution must be detected to verify its effectiveness. Therefore, the criterion proposed is used for verification, that is, the resolved scale metric should be ≤80% of the sum of the resolved kinetic energy *K* and the sub-grid kinetic energy *k*. The resolved kinetic energy *K* can be calculated by the following formula:

(9) $$K=0.5({\tilde{u}}_{x}{\tilde{u}}_{x}+{\tilde{u}}_{y}{\tilde{u}}_{y}+{\tilde{u}}_{z}{\tilde{u}}_{z})$$

In Equation (9): ${\tilde{u}}_{x}$, ${\tilde{u}}_{y}$, ${\tilde{u}}_{z}$—fluctuation velocities in the x, y, and z directions.

Meanwhile, the fluctuations of the *Sub-Grid Scale* (SGS) stress in the LES model can significantly reduce the efficiency of simulation analysis. Additionally, SGS determines the influence of the unresolved scale quantities on the momentum transfer process and its dissipation. Its definition formula is

(10) $$\tau_{sgs}=\bar{uu}-\bar{u}\;\bar{u}$$

In Equation (10): *u*—instantaneous resolved velocity field.

This study aims to close the sub-grid-scale (SGS) by adding a sub-grid-scale eddy viscosity model to improve the efficiency of simulation analysis.

(11) $${\overset{=}{\tau }}_{sgs}+\mu_{sgs}(\nabla \bar{u}+\nabla {\bar{u}}^{T})=1.5kI$$

In Equation (11): $\mu_{sgs}$—SGS turbulent viscosity, which can be calculated using a one-equation eddy-viscosity model as follows. Meanwhile, the sub-grid kinetic energy *k* can also be solved to complete the validation of grid effectiveness.

(12) ∇[(v+vsgs)∇k]−∂k∂t−∇(ku¯)=0.5τsgs(∇u¯+∇u¯T)−ε

In Equation (12): ε—turbulent dissipation; $v_{sgs}$—SGS kinematic viscosity; which can be calculated as follows:

(13) ε=Cεk3/2Δ

(14) $$v_{sgs}=C_{k}k^{1/2}\Delta$$

In Equations (13) and (14): Δ—SGS grid size, $\Delta =V^{1/3}$; *V*—volume of the computational cell under consideration; $C_{\varepsilon }$ = 1.048; $C_{k}$ = 0.094.

By programming and embedding the above equations to replace the original LES model in the Fluent module, the N-LES turbulence model suitable for the analysis of the Baijiu aging process can be obtained. The N-LES turbulence model can not only combine the resolvable scale quantity ≤ 0.8 (*K* + *k*) to complete the grid validity test for accurate grid division but also achieve the closure of the SGS, thereby improving the efficiency of simulation analysis.

#### 3.2.3. Optimization of the VOF Sub-Model

Considering that the traditional VOF model assumes that the velocities and pressures of the gas–liquid two-phase are consistent within the same grid and only solves a single momentum equation, which weakens the resistance caused by the velocity difference between the gas and liquid phases at the phase interface, it thus shows a large difference from the actual cavitation and explosion process of the wine body. The authors optimized the traditional VOF model as follows based on Weller’s interface compression method.

(1) Optimization of continuity equation

We added an “artificial” compression term to the convection term ($\nabla \rho_{i}\alpha_{i}u_{i}$) in the continuity equation (as shown in Equation (15)) that each phase in the traditional VOF model must satisfy to sharpen the phase interface and enhance the interface capture ability, as shown in Equations (16)–(19).

(15) ∂(ρiαi)∂t+∇(ρiαiui)=M

(16) $$\nabla (\rho_{i}\alpha_{i}u_{i})=\nabla (\rho_{i}\alpha_{i}U)+\nabla \left[\sum_{\begin{array}{l} j=1\\ i\neq j \end{array}}^{n}\rho_{i}\alpha_{i}\alpha_{j}(u_{i}-u_{j})\right]$$

(17) $$(u_{i}-u_{j})=C_{a}\left|U\right|n$$

(18) $$U=\sum_{i=1}^{n}a_{i}u_{i}$$

(19) n=(αj∇αi−αi∇αj)αj∇αi−αi∇αj

In Equations (15)–(19): $\rho_{i}$—density of the *i* phase; $\alpha_{i}$—volume fraction of the *i* phase; $u_{i}$—instantaneous velocity of the *i* phase; *M*—mass source phase for mass transfer between two phases; $\nabla \left[\sum_{\begin{array}{l} j=1\\ i\neq j \end{array}}^{n}\rho_{i}\alpha_{i}\alpha_{j}(u_{i}-u_{j})\right]$—“artificial” compression term, where $\alpha_{i}\alpha_{j}$ ensures that this “artificial” compression term only acts at the phase interface. After the diffused free interface is compressed, it becomes thinner and the phase interface is sharpened. $C_{a}$—interface compression factor. When set to 0, the model will not perform interface compression and capture on the diffused surface; when set to 1, it will perform all-around interface compression and capture on the diffused surface, increasing steplessly from 0 to 1. To obtain the appropriate results, it can be adjusted between 0.2 and 0.9 according to the actual situation. $\left|U\right|$—magnitude of the phase-averaged velocity, used to control the magnitude of the compression velocity of $u_{i}-u_{j}$. *n*-normal vector of the interface element, used to define the direction of the compression velocity.

(2) Optimization of the *Schnerr–Sauer* equation

In the actual process of the Baijiu body cavitation–explosion, the bubble number density *J* is a variable closely related to the mass flow rate, contact angle *θ*, saturation vapor pressure, and local pressure difference. However, in the *Schnerr–Sauer* equation of the traditional VOF model, *J* is usually set as a constant value based on experience, and thus cannot accurately describe the cavitation–explosion process of the Baijiu body. Therefore, in this study, *J* is corrected based on the heterogeneous nucleation theory as follows.

(20) J=N02/3ψ(BThp)exp−16πσ3ω3BT(pv−p)2

(21) ψ=0.5+0.5cosθ

(22) $$\omega =0.25{\left(1+\cos\theta \right)}^{2}(2-\cos\theta )$$

In Equations (20)–(22): *N*_0_—number of molecules per unit volume; *B*—Boltzmann constant; *T*—nucleation temperature; *σ*—surface tension coefficient; *h_p_*—Planck constant; *p_v_*—saturated vapor pressure; *ψ*—surface area available for heterogeneous nucleation in the liquid phase per unit volume; *ω*—geometric correction factor for the minimum work required to form a critical nucleus; *θ*—contact angle.

By substituting the above equations into the original traditional VOF model in the Fluent module, the N-VOF model is optimized. By replacing the above optimized conservation equations, the N-LES large eddy model, and the N-VOF model into the corresponding modules in Fluent, the optimized N-CFD + VOF model is obtained. By importing the physical model, setting the boundary conditions as shown in Table 1, and starting the N-CFD + VOF model in ANSYS-Fluent 2024, the simulation analysis of the cavitation–explosion process of Baijiu can be completed [23,24].

### 3.3. Optimization Design of Key Structures

#### 3.3.1. Optimization of Nozzle Inner Diameter

**Figure 6:**
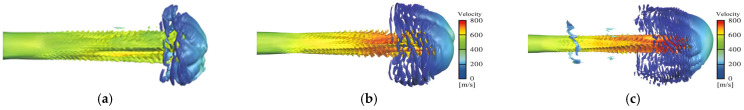
Velocity distribution of high-speed jets under different inner diameters. ((**a**) *d*_1_ = 9 mm; (**b**) *d*_2_ = 8 mm; (**c**) *d*_3_ = 7 mm).

**Figure 7:**
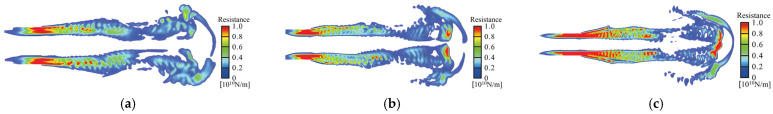
Depiction of the resistance distribution of high-speed jets under different inner diameters. ((**a**) *d*_1_ = 9 mm; (**b**) *d*_2_ = 8 mm; (**c**) *d*_3_ = 7 mm).

First, according to the design requirement of the nozzle inner diameter *d* = 7–9 mm, physical models with inner diameters of *d*_1_ = 9 mm, *d*_2_ = 8 mm, and *d*_3_ = 7 mm are established step-by-step at an interval of 1 mm. Then, the boundary conditions are set as shown in Table 1 and imported into the physical models, respectively. The optimized N-CFD + VOF model in Fluent is used for simulation analysis. The velocity and resistance distributions of the high-speed jet after passing through the nozzle are obtained, as shown in Figure 6 and Figure 7, respectively.

As shown in Figure 6, with the decrease in the nozzle inner diameter, the velocity of the jet body increases sharply, but the diameter, as well as the amplitude and wavelength of the surface waves formed by “cavitation”, all decrease. This indicates that within the range of 7–9 mm, the smaller the inner diameter, the less conducive it is for the formation of cavitation bubbles in Baijiu through “cavitation”. However, at the same time, the number of broken droplets at the trailing edge formed by the “violent explosion of cavitation bubbles” at the jet head increase significantly, and the droplets can be detached from the jet head more quickly and continue to explode. This shows that within the range of 7–9 mm, the smaller the inner diameter, the more conducive it is for the “explosion–catalysis of cavitation bubbles”. As shown in Figure 7, with the decrease in the nozzle inner diameter, the shear force between the jet and the air becomes stronger. In particular, the drag force acting on the jet head also increases sharply, which theoretically is more conducive to the “violent explosion–catalysis of cavitation bubbles” at the jet head. However, at the same time, the shear force acting on the trailing section of the jet body also increases significantly, which is obviously unfavorable for the formation of cavitation bubbles in the jet body inside the nozzle through “cavitation”.

In summary, to reconcile the conflicting requirements—larger nozzle diameters favoring the generation of more bubbles for enhanced cavitation, while smaller diameters promoting more intense bubble collapse for superior aging of Baijiu—this study ultimately selected a nozzle inner diameter of *d*_2_ = 8 mm for the new Baijiu maturation device.

#### 3.3.2. Optimization of the Distance Between the Reaction Chamber Walls

**Figure 8:**
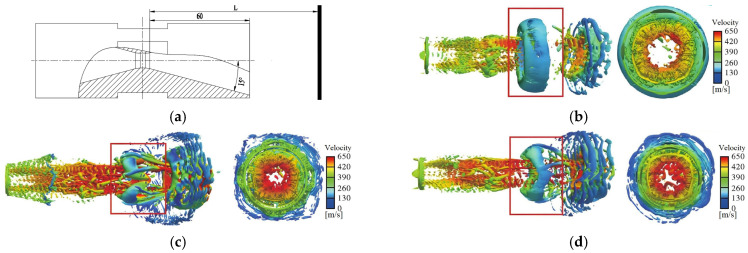
Eddy current distributions generated by high-speed jets with different inner diameters. ((**a**) Definition of *L*; (**b**) *L*_1_ = 130 mm; (**c**) *L*_2_ = 150 mm; (**d**) *L*_3_ = 170 mm).

A nozzle model with an inner diameter of *d*_2_ = 8 mm was used, and the variable was set as the distance between the nozzle and the reaction chamber wall (as shown in Figure 8a), specifically *L*_1_ = 130 mm, *L*_2_ = 150 mm, and *L*_3_ = 170 mm. Then, referring to Table 1 to set the boundary conditions, the flow of the jet formed by a single nozzle in the reaction chamber was analyzed. The distribution of the obtained flow field (turbulent vortices) is shown in Figure 8.

As shown in Figure 8, the vortex structures uniformly distributed in the main body of the jet after leaving the nozzle do not differ significantly, indicating that *L* has a relatively small influence on the “cavitation” of Baijiu. However, the vortex structures in the umbrella-shaped fragmentation zone at the jet head do vary greatly, suggesting that *L* has a significant impact on the “catalytic effect of intense bubble bursting”. When *L*_1_ = 130 mm, cracks start to appear in the axisymmetric annular vortex at the jet neck, but the cracks have not freely fragmented to cause the complete “free and intense bursting” of bubbles. Immediately afterwards, the vortex collides with the inner wall of the reaction chamber and breaks. As the distance increases to *L*_2_ = 150 mm, under the continuous action of resistance, the axisymmetry of the turbulent vortices in the umbrella-shaped fragmentation zone at the jet head is broken, causing the jet neck to fragment and form a large number of tiny turbulent vortices, leading to the complete “free and intense bursting” of bubbles. Compared with the situation at *L*_2_ = 150 mm, when *L*_3_ = 170 mm, the umbrella-shaped fragmentation zone at the jet head is significantly reduced, and the number of tiny turbulent vortices around it also decrease sharply, resulting in excessive “free and intense bursting” of bubbles. Therefore, when implementing market-oriented production, the distance *L* between the nozzle and the wall of the reaction chamber can be selected to be in the range of 140–160 mm according to the model (overall size) of the aging equipment.

#### 3.3.3. Simulation Analysis of Chinese Baijiu Aging Process

**Figure 9:**
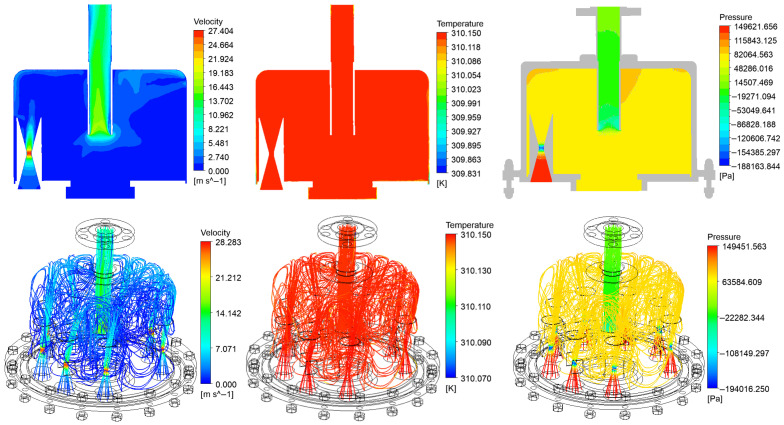
Pressure, temperature and process distribution of the liquor aging process.

Taking the most core catalytic reaction area in the Chinese Baijiu aging process, namely the “nozzle + reaction chamber”, as a whole, the N-CFD + VOF model was used for simulation analysis, and the distributions of its velocity, temperature and pressure field are shown in Figure 9.

## 4. Experimental Verification

**Figure 10:**
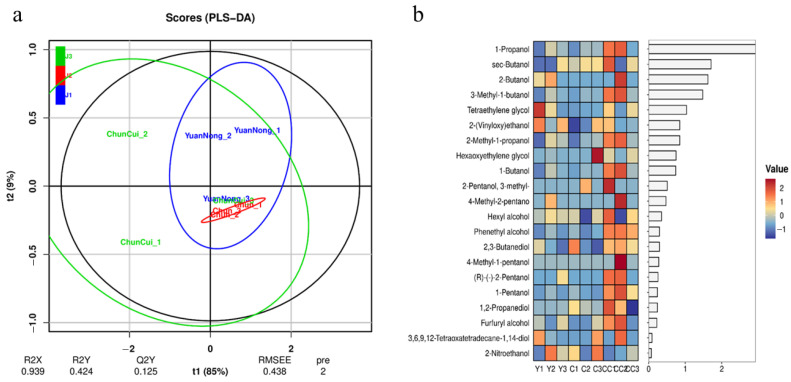
OPLS-DA analysis of alcohols in wine samples and heat map of the main differential substances therein. ((**a**) OPLS-DA analysis chart; (**b**) heat map).

**Figure 11:**
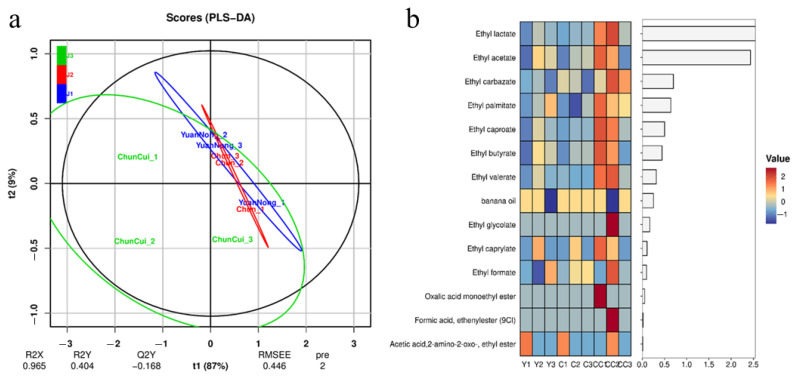
OPLS-DA analysis of lipids in wine samples and heat map of the main differential substances therein. ((**a**) OPLS-DA analysis chart; (**b**) heat map).

**Figure 12:**
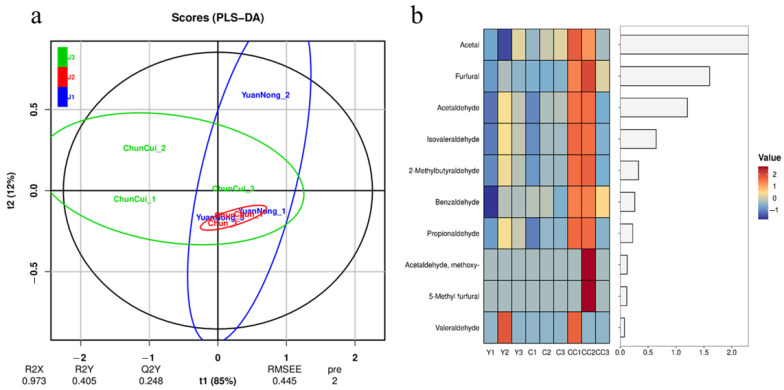
OPLS-DA analysis of aldehydes in wine samples and heatmap of the main differential substances therein. ((**a**) OPLS-DA analysis chart; (**b**) heat map).

Place 100 L of Baijiu into a new-type physical aging equipment and conduct aging for 15 min with parameters set as shown in Table 1. Immediately afterwards, label the Baijiu “stored in steel tanks for 15 days”, the Baijiu “stored in ceramic jars for 15 days” and the Baijiu “physically aged + stored in ceramic jars for 15 days” as samples J1, J2 and J3, respectively. Then, use gas chromatography–mass spectrometry (GC-MS) to sequentially measure the physicochemical indicators such as the contents of alcohols, esters and aldehydes in samples J1, J2 and J3 [25,26,27]. Finally, perform OPLS-DA analysis, and the results are shown in Figure 10a, Figure 11a and Figure 12a. Select substances with larger VIP values as the main differential substances for analysis, and the heat maps of the obtained main differential substances are shown in Figure 10b, Figure 11b and Figure 12b, where the labels Y, C and CC in the Figures correspond to J1, J2 and J3, respectively.

Among them, the procedures for detection by gas chromatography–mass spectrometry (GC-MS) are as follows [13,28]. ① Sample preparation: Dilute the sample with an ethanol–water solution (e.g., 50% *v*/*v*) and add an internal standard (4-methyl-2-pentanol or ethyl heptanoate) to ensure accurate quantification. ② Employ headspace solid-phase microextraction (HS-SPME) technology to enhance the sensitivity to trace aldehydes. ③ Use a medium-polarity capillary column (DB-WAX, 60 m × 0.25 mm × 0.25 μm) with a programmed temperature to achieve separation. ④ Compare the mass spectra of the eluted compounds with the reference library and compare the retention indices with those of the standards. Quantify using the internal standard method based on the calibration curves established for each target analysed. The main experimental reagents and equipment are shown in Table 2 below.

As shown in Figure 10a, the concentrations of J1 and J2 are relatively high with small differences between the groups, while J3 is more dispersed compared to J1 and J2. This indicates that the differences in alcohol substances between J1 and J2 are not significant, while the differences in alcohol substances between J3 and J1, J2 are relatively significant. As shown in Figure 10b, among the main differential alcohol substances, the contents of n-propanol, 2-butanol, and isoamyl alcohol in J3 are higher than those in J1 and J2. However, the content of methanol in J3 was significantly lower than that in J1 and J2. Other alcohols have little effect on flavor, so it is only necessary to conduct safety analysis.

As shown in Figure 11a, the concentrations of J1 and J2 are relatively high with small differences between the groups, while J3 is more dispersed compared to J1 and J2. This indicates that the differences in ester substances between J3 and J1 and J2 are relatively obvious, while the differences in ester substances between wine samples J1 and J2 are not significant. As shown in Figure 11b, the contents of methyl acetate, ethyl acetate and ethyl lactate in J3 were significantly higher than those in J1 and J2. However, the contents of ethyl formate, ethyl palmitate, ethyl hexanoate, ethyl-butyrate and ethyl valerate increased slightly. Other aldehydes have little effect on flavor, so it is only necessary to conduct safety analysis.

As shown in Figure 12a, the concentrations of J1 and J2 are relatively high with small differences between groups, while J3 is more dispersed compared to J1 and J2. This indicates that the differences in aldehyde substances between J3 and J1 and J2 are relatively obvious, while the differences in aldehyde substances between J1 and J2 are not significant. As shown in Figure 12b, the content of acetal, furfural and acetaldehyde in J3 is significantly higher than that in J1 and J2, and the content of Acetal in J2 is also higher than that in J1. At the same time, the content of other aldehydes in J1, J2 and J3 has no significant change. Other lipids have little effect on flavor, so it is only necessary to conduct safety analysis.

By analyzing the content changes in the above-mentioned alcohols, esters, and aldehydes in J1, J2, and J3, it can be found that compared with “storage in steel tanks for 15 days”, “storage in ceramic jars” for 15 days only increased the content of acetal in the Baijiu, while the contents of other alcohols, esters, and aldehydes did not change significantly. However, compared with “storage in ceramic jars” for 15 days, after “physical aging + storage in ceramic jars” for 15 days, the contents of alcohols, esters, and aldehydes in the Baijiu changed significantly. Specifically, the contents of n-propanol, 2-butanol, isoamyl alcohol, ethyl lactate, ethyl acetate, methyl acetate, acetal, furfural, acetaldehyde, etc., increased significantly, while the content of methanol decreased significantly. Among them, ethyl lactate, ethyl acetate, methyl acetate, acetal, and furfural, whose contents increased significantly, are the main sources of the floral, fruity, and caramel aromas of Baijiu, and acetaldehyde and n-propanol are the main components that enhance the aroma release of Baijiu [13,29,30]. Meanwhile, the reduced methanol is the main source of the sour and astringent taste of Baijiu. Specifically, the concentrations of ethyl acetate, ethyl lactate, and ethyl palmitate increased from 6.105 ± 0.014, 3.498 ± 0.015 mg/L and 0.621 ± 0.010 mg/L to 6.332 ± 0.016, 4.868 ± 0.012 mg/L and 0.681 ± 0.008 mg/L, respectively, and the concentration of methanol decreased by 68.14% ± 2.25%.

In conclusion, in the new physical aging process and equipment, the instantaneous high-temperature and high-pressure catalytic effect generated by repeatedly experiencing “cavitation instantaneous and violent bubble explosion” can greatly accelerate the rapid dehydration condensation reaction between methanol, ethanol and acetic acid and lactic acid present in Baijiu, forming three types of flavor substances: ethyl lactate, ethyl acetate, and methyl acetate [31,32]. Ultimately, it can achieve efficient, low-cost, additive-free, and healthy aged Baijiu, thereby reducing the storage cost and risk of Baijiu production volume and promoting the high-speed and healthy development of the Baijiu industry.

## 5. Conclusions

In this paper, based on the characteristics that the pressure change, temperature change, cavitation, etc., caused by a high-speed jet of Baijiu can catalyze the reaction of components such as water, ethanol, and acetic acid to form unique flavor substances, a set of additive-free, healthy, and efficient physical aging processes and equipment were developed. Then, an N-CFD + VOF model was established to achieve accurate simulation analysis of the physical aging process of Baijiu, and the key structural optimization design of the nozzle and reaction chamber, the core components of the aging equipment, was completed. Finally, through the application in distilleries and the detection of key indicators, the correctness of the new physical aging process of Baijiu was verified, and the mechanism of the change in internal components in the Baijiu during the physical aging process was revealed.

Through the 15 min aging test of 100 L multi grain Baijiu, the concentration of methanol in Baijiu decreased by 68.14 ± 2.25%, and the concentrations of ethyl acetate, ethyl lactate and ethyl palmitate increased from 6.105 ± 0.014, 3.498 ± 0.015 mg/L and 0.621 ± 0.010 mg/L to 6.332 ± 0.016, 4.868 ± 0.012 mg/L and 0.681 ± 0.008 mg/L, respectively, which was similar to the changes in Baijiu composition after 2.5 years of storage in traditional pottery pots. At the same time, after the evaluation of enterprise experts, it was concluded that the taste and flavor of Baijiu stored in a traditional pottery jar for 2 years were consistent. In conclusion, the use of this aging process can shorten the storage and aging time of Baijiu from 2 years to 15 min, greatly reduce the cost of capital backlog, and reduce the probability of inventory fire, so as to promote the high-quality development of the Baijiu industry.

Limited by the difference between the requirements of alcohol, aldehyde and ester content detection in the process of enterprise market promotion and application and the requirements of school scientific research, this research only provided the average data of all component contents after detection, whereas we did not conduct variance analysis on all component contents; instead, only the content data of key components identified after analysis were supplemented and analyzed by variance. However, the data are measured after the large-scale production of the enterprise is stable, and the accuracy of the data and the repeatability of the experiment are guaranteed. In the follow-up study, on the one hand, we can supplement the determination of the content of non-key components for analysis of variance, while on the other hand, we can also increase the detection range of liquor components to further explore its aging mechanism.

## Figures and Tables

**Table 1:** Initial boundary conditions.

Parameter	Value	Parameter	Value
Baijiu density (kg/m^3^)	950	Air density (kg/m^3^)	1.205
Baijiu viscosity (Pa/s)	1.46 × 10^−5^	Air viscosity (Pa/s)	1.46 × 10^−11^
Baijiu specific heat capacity (KJ/(kg·K))	3.8	Air specific heat capacity (KJ/(kg·K))	1.005
Component diffusion coefficient (m^2^/s)	10^−9^	Baijiu surface tension (N/m)	0.028
Thermal conductivity (w/(m·K))	0.25	Baijiu steam viscosity (Pa/s)	1.96 × 10^−5^
Thermal conductivity (MPa)	1	Outlet pressure (MPa)	0.1

**Table 2:** Main experimental reagents and equipment.

Equipment Name	Model	Manufacturer
Gas chromatography–mass spectrometry	8890 GC/5977B MSD	Agilent Technology Co., Ltd.; Santa Clara, CA, USA.
Capillary column	DB-17ms	Agilent Technology Co., Ltd.; Santa Clara, CA, USA.
Ethyl heptanate	E305454	Aladdin; Shenzhen, China.
SPME automatic injection handle	57331	Supelco; Chicago, IL, USA.
SPME extraction head	57328-U	Supelco; Chicago, IL, USA.

## Data Availability

The original contributions presented in this study are included in the article. Further inquiries can be directed to the corresponding authors.

## References

[1] Xiong F.K., Hu G.Z., Liu Z.B., Xiao H.W., Huang B. (2025). Investigation of Damage Mechanism in Baijiu Yeast during Spray Drying at the Molecular Scale. J. Agric. Food Chem..

[2] Xiong F.K., Liu Z.B., Hu G.Z., Zhao D., Qu D.Q. (2025). Optimisation of the Protection Process in Baijiu Yeast Spray Drying and Investigation of Its Micro-Nano Protection Mechanism. LWT-Food Sci. Technol..

[3] Han B.L., Ren X.H., Gong H.C., Zhang S.F., Zhou W.T., Wei Y.H., Fang Y.L., Xu Q., Bian M.H. (2025). Effect of Compound Aging Treatment on the Quality of Nongxiangxing Baijiu. Lwt-Food Sci. Technol..

[4] He Y.X., Chen S., Tang K., Qian M., Yu X.W., Xu Y. (2021). Sensory Characterization of Baijiu Pungency by Combined Time-Intensity (TI) and Temporal Dominance of Sensations (TDS). Food Res. Int..

[5] Fan C.M., Shi X., Pan C.M., Zhang F.L., Zhou Y.Y., Hou X.G., Hui M. (2024). GC-IMS and GC/Q-TOFMS Analysis of Maotai-Flavor Baijiu at Different Aging Times. Lwt-Food Sci. Technol..

[6] Jiang Q.X., Chen M.J., Guo W., Hu X.J., Zhou W.Q., Zhao F., Xie L.L., Yang H.L. (2025). Investigating the Effect of the Particle Size of Potter’s Clay on the Pore Structure of Pottery. Jom.

[7] Yang H.L., Hu X.J., Tian J.P., Xie L.L., Chen M.J., Huang D. (2025). Exploring the Influence of Pottery Jar Formula Variables on Flavor Substances through Feature Ranking and Machine Learning: Case Study of Maotai-Flavored Baijiu. Foods.

[8] Fan G.S., Fu Z.L., Teng C., Liu P.X., Wu Q.H., Rahman M.K.R., Li X.T. (2020). Effects of Aging on the Quality of Roasted Sesame-Like Flavor Daqu. Bmc Microbiol..

[9] Du J.Y., Xu Z.X., Sun H.B., Zhu Y.F., Zhang J., Huang M.Q., Liu Y., Liu H.Q., Sun B.G., Wu J.H. (2025). Effect of a New Sea-Aging Method on the Flavor of Baijiu. Food Res. Int..

[10] Jiang S., Dai W., Chen X.P., Tan W.J., Wang J.L., Yu Y.G., Zheng Q. (2025). Vintage Authentication Technology for Aged Baijiu: Acid-Ester Equilibrium. Curr. Res. Food Sci..

[11] Wang R.J., Yan L.J., Ding F., Xu S.S., Hu W.Q., Zhang L., Xu B.Y., Zhou H., Mu D.D., Li X.J. (2025). From Mung Bean to Minglv-Flavor Baijiu: Unraveling the Signature Aroma Profile and Aging Dynamic. Food Chem..

[12] Wei J., Li Y.L., An D., Fan Z.B., Zhang R., Shi L., Luo C.X., Feng K.Y., Chang J., Chu X.G. (2020). Foodomics Analysis of Natural Aging and Gamma Irradiation Maturation in Chinese Distilled Baijiu by UPLC-Orbitrap-MS/MS. Food Chem..

[13] Wang L.L., Gao Y.C., Wu L., Chen S., Xu Y. (2024). Characterization of Key Aging Aroma Compounds in Aged Jiangxiangxing Baijiu and Their Formation Influencing Factors during the Storage Process. J. Agric. Food Chem..

[14] Zhu K.X., Xiao Q., Yuan S.Q., Liu J., Shang H.W., Guo M.Y., Zhao J.S. (2025). The Relationship between Microbial Diversity and the Physicochemical Characteristics of Pit Mud of Strong-Flavor Baijiu. Sci. Rep..

[15] Mao F.J., Huang J., Zhou R.Q., Qin H., Zhang S.Y., Cai X.B., Qiu C.F. (2023). Succession of Microbial Community of the Pit Mud under the Impact of Daqu of Nongxiang Baijiu. J. Biosci. Bioeng..

[16] Zhang H.M., Meng Y.J., Wang Y.M., Wang Y.L., Zhou Q.W., Li A.J., Liu G.Y., Li J.X., Xing X.H. (2020). Prokaryotic Communities in Multidimensional Bottom-Pit-Mud from Old and Young Pits Used for the Production of Chinese Strong-Flavor Baijiu. Food Chem..

[17] Wei J., Fan Z.B., An D., Shi L. (2022). Molecular Mechanism of Mare Nectaris and Magnetic Field on the Formation of Ethyl Carbamate during 19 Years Aging of Feng-Flavor Baijiu. Food Chem..

[18] Zheng Q., Tian W.H., Wang S.S., Liu X.L., Kong Q.L., Yue L., Yan W.Q., Zhang Y., Nong L., Xu X.Y. (2025). Using GC-IMS and FTIR to Investigate the Effect of Ionizing Radiation on Volatile Compounds and Hydrogen Bonding of Strong-Flavor Baijiu. Food Chem.-X.

[19] Dai J.H., Tang W.P., Wang Y.N., Gan X., Yang L., Zhang J., Sun Y.C., Wang Y.J., Qin H.J., Wang S.P. (2025). Investigating the Influence of 60Co Irradiation on the Aging Aroma Components of Soy Sauce Aroma Type Baijiu by Integrating E-Nose, GC-MS, GC-IMS, and Chemometric Methods. Food Chem.-X.

[20] Li X.F., Dai W., Wang J.L., Yu Y.G., Zheng Q. (2025). Thermodynamic Equilibrium-Driven Blending Technology for Baijiu Standardization. Food Chem..

[21] Geleynse S., Jiang Z.H., Brandt K., GarciaPerez M., Wolcott M., Zhang X. (2020). Pulp Mill Integration with Alcohol-to-Jet Conversion Technology. Fuel Process. Technol..

[22] Jiang X.Y., Liu D.F., Yang S.Z., Cheng X., Xie Y.Q. (2024). Evolution of Self-Assembled Amphiphilic Colloidal Particles in Strong-Flavor Chinese Baijiu. Food Chem..

[23] Huang Z.J., Zeng Y.H., Sun Q.Y., Zhang W.H., Wang S.T., Shen C.H., Shi B. (2022). Insights into the Mechanism of Flavor Compound Changes in Strong Flavor Baijiu during Storage by Using the Density Functional Theory and Molecular Dynamics Simulation. Food Chem..

[24] Wang Z., Wei J.W., Wang Y., Zhu T.T., Huang M.Q., Wu J.H., Xu Y.Q., Zhang J.L., Wang B.W. (2021). A New Method to Predict the Content Changes of Aroma Compounds during the Aging Process of Niulanshan Baijiu Using the GM (1,1) Gray Model. Flavour Fragr. J..

[25] Zhang B., Zheng S.M., Huang M.Q., Wu Q., Dong W., Wu J.H., Liu H.Q., Zhao D.R., Yu Y.G., Li J.C. (2024). Analysis of Volatile Compounds in Xiangjiao Baijiu from Different Storage Containers and Years Based on HS-GC-IMS and DI-GC-MS. Food Chem.-X.

[26] Fang C., Zhuang X.T., Li Z.G., Zou Y.F., Pu J.Z., Wang D., Xu Y. (2025). LC-MS/MS-Based Determination and Optimization of Linoleic Acid Oxides in Baijiu and Their Variation with Storage Time. Metabolites.

[27] Gao M.X., Han X.L., Yang Y.F., Wang D.L. (2025). Characterization of Volatile Compounds in Aged Jiangxiangxing Baijiu by GC × GC-TOFMS and Means of the Sensomics Approach. Food Chem..

[28] Sun X.Z., Qian Q.Q., Xiong Y.Q., Xie Q.Q., Yue X.X., Liu J.H., Wei S.X., Yang Q. (2022). Characterization of the Key Aroma Compounds in Aged Chinese Xiaoqu Baijiu by Means of the Sensomics Approach. Food Chem..

[29] Huang H., Gao Y.C., Wang L.L., Yu X.W., Chen S., Xu Y. (2024). Maillard Reaction Intermediates in Chinese Baijiu and Their Effects on Maillard Reaction Related Flavor Compounds during Aging. Food Chem. X.

[30] Wang S.Y., Li C., Li Y.J., Liu G.Q., Lu Z.M., Chai L.J., Xu H.Y., Shi J.S., Wang S.T., Shen C.H. (2025). Evolution of Aroma Compounds in Round Soy Sauce Aroma Type Baijiu during Aging and the Effect of Aging Markers on the Lasting Aroma in Finished Glass. Food Chem.-X.

[31] Qiu W., Ru H.L., Wang J.L., Kuang J.M., Yu Y.G., Zheng Q. (2025). Odor Threshold Dynamics during Baijiu Aging: Ester-Acid Interactions. Lwt-Food Sci. Technol. Technol..

[32] Wu Y.S., Chen H., Sun Y., Huang H., Chen Y.Y., Hong J.X., Liu X.X., Wei H.Y., Tian W.J., Zhao D.R. (2023). Integration of Chemometrics and Sensory Metabolomics to Validate Quality Factors of Aged Baijiu (Nianfen Baijiu) with Emphasis on Long-Chain Fatty Acid Ethyl Esters. Foods.

